# Safety of continuous intraoperative vagus nerve neuromonitoring during thyroid surgery

**DOI:** 10.1093/bjsopen/zrad039

**Published:** 2023-06-08

**Authors:** Timothy Mathieson, Wedali Jimaja, Frédéric Triponez, Marc Licker, Wolfram Karenovics, Petra Makovac, Mirza Muradbegovic, Valentina Belfontali, Benoît Bédat, Marco Stefano Demarchi

**Affiliations:** Department of Thoracic and Endocrine Surgery and Faculty of Medicine, University Hospitals of Geneva, Geneva, Switzerland; Department of Thoracic and Endocrine Surgery and Faculty of Medicine, University Hospitals of Geneva, Geneva, Switzerland; Department of Thoracic and Endocrine Surgery and Faculty of Medicine, University Hospitals of Geneva, Geneva, Switzerland; Department of Anaesthesiology and Faculty of Medicine, University Hospitals of Geneva, Geneva, Switzerland; Department of Thoracic and Endocrine Surgery and Faculty of Medicine, University Hospitals of Geneva, Geneva, Switzerland; Department of Thoracic and Endocrine Surgery and Faculty of Medicine, University Hospitals of Geneva, Geneva, Switzerland; Department of Thoracic and Endocrine Surgery and Faculty of Medicine, University Hospitals of Geneva, Geneva, Switzerland; Department of Thoracic and Endocrine Surgery and Faculty of Medicine, University Hospitals of Geneva, Geneva, Switzerland; Department of Thoracic and Endocrine Surgery and Faculty of Medicine, University Hospitals of Geneva, Geneva, Switzerland; Department of Thoracic and Endocrine Surgery and Faculty of Medicine, University Hospitals of Geneva, Geneva, Switzerland

## Abstract

**Background:**

Continuous intraoperative neuromonitoring has successfully demonstrated to predict impending damage to the recurrent laryngeal nerve, by detecting changes in electromyographic recordings. Despite the apparent benefits associated with continuous intraoperative neuromonitoring, its safety is still a debate. The aim of this study was to investigate the electrophysiological impact of continuous intraoperative neuromonitoring on the vagus nerve.

**Methods:**

In this prospective study, the amplitude of the electromyographic wave of the vagus nerve–recurrent laryngeal nerve axis was measured both proximally and distally to the stimulation electrode placed upon the vagus nerve. Electromyographic signal amplitudes were collected at three distinct events during the operation: during the dissection of the vagus nerve, before application of the continuous stimulation electrode onto the vagus nerve and after its removal.

**Results:**

In total, 169 vagus nerves were analysed, among 108 included patients undergoing continuous intraoperative neuromonitoring-enhanced endocrine neck surgeries. Electrode application resulted in a significant overall decrease in measured proximo-distal amplitudes of −10.94 µV (95 per cent c.i. −17.06 to −4.82 µV) (*P* < 0.005), corresponding to a mean(s.d.) decrease of −1.4(5.4) per cent. Before the removal of the electrode, the measured proximo-distal difference in amplitudes was −18.58 µV (95 per cent c.i. −28.31 to −8.86 µV) (*P* < 0.005), corresponding to a mean(s.d.) decrease of −2.50(9.59) per cent. Seven nerves suffered a loss of amplitude greater than 20 per cent of the baseline measurement.

**Conclusion:**

In addition to supporting claims that continuous intraoperative neuromonitoring exposes the vagus nerve to injury, this study shows a mild electrophysiological impact of continuous intraoperative neuromonitoring electrode placement on the vagus nerve–recurrent laryngeal nerve axis. However, the small observed differences are negligible and were not associated with a clinically relevant outcome, making continuous intraoperative neuromonitoring a safe adjunct in selected thyroid surgeries.

## Teaser

Continuous intraoperative neuromonitoring has been successfully demonstrated to predict damage to the recurrent laryngeal nerve by detecting changes in electromyographic recordings, but continuous intraoperative neuromonitoring exposes the vagus nerve to potential injury. This prospective study, which enrolled 169 nerves at risk, shows that continuous intraoperative neuromonitoring electrode placement causes a very mild yet statistically significant electrophysiological impact on the vagus nerve, although without any clinically relevant impact.

## Introduction

The overall incidence of transient or permanent recurrent laryngeal nerve (RLN) palsy after thyroid surgery is low (5–8 and 0.3–3 per cent respectively)^1^, but can vary according to the condition warranting surgery, the size of the gland to be removed, reoperation, and the experience of the surgeon^2–5^. The consequences of RLN palsy are associated with significant morbidity rate, with symptoms including dysphonia (80 per cent), dyspnoea during daily activities (75 per cent), dysphagia (56 per cent), and aspiration (54 per cent)^6–8^. Clinical outcomes after bilateral RLN palsy are devastating, as the functional integrity of the upper airways is compromised and affected patients likely require tracheostomy^9^. Furthermore, RLN palsy is the leading cause of litigations for malpractice claims in the field of endocrine surgery^10–12^.

The main limitation of traditional intraoperative neuromonitoring (IONM) stems from its inability to predict RLN injury, as it can only show loss of signal (LOS) once it has occurred, suggesting iatrogenic nerve damage. This constraint has led to further expansion in the field of RLN neuromonitoring, with the development of a novel continuous form of RLN stimulation, providing the surgeon with a constant display of the nerve’s conduction^13–16^. This technology involves placement of an electrode on the vagus nerve (VN), above the level of RLN branching, and periodic stimulation (10−60/min per preference) of the VN–RLN axis, with detection of sustained electromyographic (EMG) signals from the laryngeal muscles throughout surgery.

Since its first application in the last decade, continuous intraoperative neuromonitoring (CIONM) has been successfully demonstrated to predict damage to the RLN by detecting changes in EMG nerve conduction, which, when correlated with a specific surgical manoeuvre (mainly traction on the nerve), informs the surgeon of mechanical insult to the nerve tissue, putting the RLN at risk of impending injury^17–19^. Impending nerve damage is signalled to the surgeon by a ‘combined event’ (CE), defined as a combination of drop in EMG signal amplitude greater than or equal to 50 per cent and an increase in latency greater than or equal to 10 per cent^17^. CEs are considered a precursor to complete LOS at a still reversible stage, alerting the surgeon and providing time for protective measures^14,17,20^.

Ongoing research suggests that early detection of alterations in the electrophysiology of the VN–RLN axis may lead to a decreased incidence of RLN palsy by enabling the surgeon to release tissue or correct a manoeuvre that causes stress to the nerve^17,18,20,21^. Recently, Schneider *et al*.^22^ conducted a retrospective cohort study of 6029 patients and found that the use of CIONM compared with intermittent intraoperative neuromonitoring (IIONM) alone was associated with 1.8 times fewer transient vocal cord palsies and 29.4 times fewer frequent permanent vocal cord palsies.

In recent years, there has been increasing interest in CIONM in the field of thyroid surgery, and many institutions, including the authors', use this technique routinely in high-risk or complex thyroid or parathyroid surgeries. Despite the apparent advantages of this technique, CIONM has not been implemented in the practice of most surgeons. There are a few explanations for the lack of CIONM-assisted interventions, such as the added time and skill required for VN dissection and application of the electrode, as well as the steep learning curve to attain full proficiency with this technology^18,23,24^. Owing to its continuous nature, the surgeon must constantly monitor the recorded signals and is often confronted with EMG changes due to artefacts caused by endotracheal tube displacement within the larynx during surgical manoeuvres. In fact, if displacement of the EMG endotracheal tube occurs, poor contact between the electrodes and vocal cords results in EMG amplitude changes^25–28^. These constraints have contributed to the limited use of CIONM among thyroid surgeons, and those who have adopted it tend to use it preferentially in cases deemed difficult^18^.

However, the main reason for its lack of use is the ongoing debate regarding safety. Some areas of concern are the potential systemic effects of increased vagal tone as a consequence of CIONM and the risk of lesions during vagal dissection or those attributable to the positioning of the monitoring apparatus itself^29^. Claims regarding the safety of VN stimulation are based on its therapeutic applications in the fields of neurology and psychiatry. VN stimulation has been FDA-approved since 1997 for cases of refractory epilepsy and since 2005 for major depression^30^. Potential therapeutic uses of VN stimulation in the management of a wide array of conditions are being studied, including obesity, neuropsychiatric disorders, chronic pain syndromes, stroke rehabilitation, and heart failure^31^. The majority of undesirable effects of VN stimulation are temporary and consist of vocal changes, dysphagia, modified respiratory patterns, paraesthesia, and pain^32^.

Another area of concern is the increased risk of trauma to the VN, both dissection-related and resulting from electrode application upon the nerve, as CIONM requires direct access and 360° dissection around the VN. Two cases of iatrogenic lesions of the VN related to dissection or application of the electrode on the VN were reported by Brauckhoff *et al*.^33^ in their prospective study. Terris *et al*.^29^ also reported a case of transient vocal cord palsy after traumatic dislodgement of the electrode with perineural ecchymosis. In the 2018 review of 101 surgeries^20^, the authors described three cases in which LOS at the level of the VN directly after application of the electrode was observed. However, to the best of the authors' knowledge, very few instances of LOS due to direct trauma to the VN have been reported. The aim of this prospective study was to address the safety concerns that have been raised with the increasing use of CIONM in thyroid and parathyroid surgery by focusing on the intraoperative impact of the application of the CIONM electrode device on the electrophysiology of the VN–RLN axis.

## Methods

All patients undergoing thyroidectomy or parathyroidectomy with CIONM were enrolled prospectively from December 2018 to March 2021. Patients with non-RLNs and those with pre-existing RLN palsy were excluded from the study.

The primary endpoint was the measurement of the amplitude of the EMG wave of the VN–RLN axis both proximal and distal to the placement of the stimulation electrode on the VN to detect any differences in nerve conduction linked to the use of CIONM. These measurements were carried out at three distinct events during the procedure: before VN dissection, before and immediately after surgical excision of the thyroid lobe and removal of the electrode. The secondary endpoints were to identify and report any adverse effects potentially due to autonomic nervous system imbalances in the setting of CIONM.

In each patient and for each individual nerve, the following parameters were assessed:

- Position of VN within the carotid sheath.
- Diameter of the selected automatic periodic stimulation (APS^®^; Medtronic Inc., Minneapolis, MN, USA) electrode.
- Number of times the APS^®^ electrode was dislodged.
- Duration of continuous VN stimulation.
- Visible damage to the VN after dissection and after APS^®^ electrode removal.
- Amplitude of the EMG response wave at specific stages of the intervention using the *L1-V1-R1-R2-V2-L2* formula, as described by the International Neural Monitoring Study Group (INMSG)^34,35^, with modifications reflecting stimulation proximal or distal to the APS^®^ electrode in order to obtain the proximo-distal amplitude gap. The formula and the authors' modifications are shown in *Table 1*. *R1* and *R2* values were also measured to exclude patients with intraoperative RLN injury from the statistical analysis of values obtained at the end of the intervention before APS^®^ electrode removal. *L1* and *L2* values were not reported as, in the authors' practice, patients are not subjected to systematic preoperative laryngoscopy and postoperative laryngoscopy is only performed in cases with significant LOS during surgery in case of vocal hoarseness after surgery.
- Cardiovascular effects during VN stimulation as reported by the anaesthesiology team.

**Table 1:** Description of the International Neural Monitoring Study Group recommendations for steps in neuromonitoring during thyroid surgery and modifications reflecting additional measurements taken in the context of the current study

INMSG step denomination*		Modified INMSG steps	
*L1*	Evaluation of preoperative vocal fold function on laryngoscopy		
*V1*	Verification of VN functional integrity before dissection	*V1.0*	Amplitude before VN dissection
		*V1.1*	Amplitude after VN dissection
		*V1.2prox*	Amplitude proximal to APS^®^ electrode, immediately after application
		*V1.2dist*	Amplitude distal to APS^®^ electrode, immediately after application
*R1*	Verification of RLN functional integrity before dissection		
*R2*	Verification of RLN functional integrity after dissection		
*V2*	Verification of VN functional integrity after dissection	*V2prox*	Amplitude proximal to APS^®^ electrode, before removal
		*V2dist*	Amplitude distal to APS^®^ electrode, before removal
*L2*	Evaluation of postoperative vocal fold function on laryngoscopy		
		*ΔV1.0-V1.1*	Difference in amplitude readings before and after VN dissection
		*ΔV1.2prox-V1.2dist*	Difference in amplitude readings proximal and distal to the APS^®^ electrode, after application
		*ΔV2prox-V2dist*	Difference in amplitude readings proximal and distal to the APS^®^ electrode, before removal

* Schneider *et al*.^24^. INMSG, International Neural Monitoring Study Group; APS, automatic periodic stimulation; RLN, recurrent laryngeal nerve; VN, vagus nerve.

The research project was submitted and approved by the local ethics committee in March 2018 (2017–01283) and all participating patients provided informed consent. The surgical team consisted of six surgeons specialized in the field of neck endocrine surgery (two experts and four fellowship trainees). All procedures were performed either by the experts or under their supervision. CIONM was selectively used for complex cases in which dissection was deemed to be associated with a high risk of RLN injury. The decision to use CIONM was made either before surgery or intraoperatively, according to the surgeon’s judgement.

All interventions were performed following an identical anaesthetic protocol. Neuromuscular transmission was monitored in the right wrist, with consecutive train-of-four (TOF) stimulations of the ulnar nerve. General anaesthesia was induced intravenously with a bolus of 10 µg sufentanyl and continuous propofol via a target-controlled infusion device to achieve a bispectral index (BIS) between 30 and 60. All patients were administered a single dose of rocuronium (0.8 mg/kg). When maximal neuromuscular blockade was achieved, a NIM-EMG endotracheal tube (Medtronic-Xomed) was placed under direct laryngoscopy with the exposed electrodes in close contact with the vocal cords. Rotation and cranial/caudal displacement of the endotracheal tube could be detected at subsequent IIONM using EMG impedance and was corrected manually for optimal positioning. No additional rocuronium was administered after the first dose.

All surgeries were performed with simultaneous use of IIONM and CIONM with a closed 2- or 3-mm diameter APS^®^ electrode depending on VN size (NIM-Response^®^ 3.0, Medtronic Inc.). Once dissection of the VN was achieved and the APS^®^ electrode applied, the system was calibrated to obtain a sufficient EMG amplitude (at least 500 µV in most cases). If the measured amplitude was insufficient, the endotracheal tube was repositioned until a satisfactory amplitude was achieved. Continuous stimulation was performed at a rate of 60/min with a current of 1 mA, following the manufacturer’s recommendations. IIONM was used with an intermittent monopolar atraumatic ball tip simulator at an initial current of 2 mA, which was usually reduced to 1 mA during the intervention after the RLN had been found. When a CE triggered an alarm, all surgical manoeuvres were stopped and the troubleshooting algorithm described by the INMSG^35^ was applied.

**Fig. 1:**
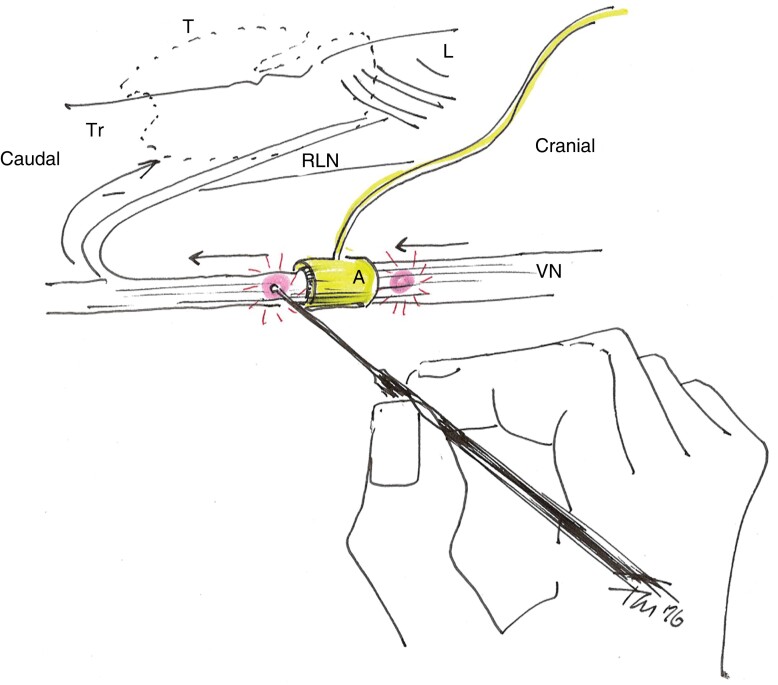
Representation of left vagus nerve stimulation proximal and distal to the automatic periodic stimulation electrode using the intermittent probe A, automatic periodic stimulation electrode; L, larynx; T, thyroid outline; Tr, trachea; VN, vagus nerve; RLN, recurrent laryngeal nerve.

For each dissected and monitored VN, EMG signal amplitudes were measured using the intermittent probe at three distinct events during the operation (before and after VN dissection, after APS^®^ electrode application, and before its removal after thyroid lobe excision), as well as the amplitude of the RLN upon identification and at the end of the intervention. After application of the APS^®^ electrode and before its removal, EMG wave amplitudes were measured both proximally and distally to the electrode in order to detect any change in amplitude, as depicted in *Fig. 1*. The differences in amplitudes are expressed as *ΔV1.0-V1.1*, *ΔV1.2prox-V1.2dist*, and *ΔV2prox-V2dist*, as shown in *Table 1*. Values obtained at different time points during the intervention, such as after application of the APS^®^ electrode and before its removal, were not paired, as external factors such as residual curarization and slight modifications of the position of the endotracheal tube during surgery could alter their comparability.

These paired values were used for statistical analysis with the Student’s two-tailed paired *t* test and expressed as a percentage of change with respect to the amplitude distal to the electrode. To identify demographic or surgical factors that may influence the measured differences in amplitude, univariable and multivariable linear regressions were performed. All statistical analyses were performed using the RStudio software (V.1.2.5033, 2009–2019, RStudio). Two-tailed *P* values <0.05 were considered statistically significant.

## Results

In total, 108 patients underwent thyroid or parathyroid CIONM-enhanced surgery between December 2018 and March 2021. Five were excluded because of insufficient data, and two did not consent to enrolment. The total number of nerves monitored was 169. The demographic and surgical characteristics of the study population, and the surgical and histopathological features for all nerves at risk are summarized in *Table 2* and*Table 3* respectively.

**Table 2:** Demographic and surgical characteristics of the study population (*n* = 101)

Variable	Value
**Age (years), median (i.q.r.)**	52 (42–66)
**Sex**	
Male	29 (29)
Female	72 (71)
**BMI (kg/m^2^), median (i.q.r.)**	26.0 (23.4–29.1)
**Preoperative diagnosis**	
Multinodular goitre	45 (45)
WDTC	19 (19)
Medullary thyroid carcinoma	1 (1)
**Intervention**	
Total thyroidectomy	68 (67)
Unilateral thyroidectomy	30 (30)
Parathyroidectomy	2 (2)
**Neck dissection (central and/or lateral)**	25 (25)

Values are *n* (%) unless otherwise indicated. i.q.r., interquartile range; WDTC, well differentiated thyroid carcinoma.

**Table 3:** Surgical and histopathological features for all nerves at risk (*n* = 169)

Variable per nerve at risk	Value
**Operative procedure**	
Thyroid lobectomy	136 (80)
Thyroid lobectomy + central neck dissection	29 (17)
Central neck dissection alone	2 (1.2)
Parathyroidectomy	2 (1.2)
**Neck dissection**	
None	138 (82)
Central	28 (17)
Lateral	3 (1.8)
**Side operated on**	
Left	85 (50)
Right	84 (50)
**Revision surgery**	7 (4.1)
**Postoperative diagnosis**	
Follicular hyperplasia	93 (55)
WDTC	55 (32.5)
Follicular adenoma	10 (5.9)
Medullary thyroid cancer	2 (1.2)
Oncocytic thyroid carcinoma	2 (1.2)
Parathyroid hyperplasia	2 (1.2)
Undifferentiated thyroid malignancy	2 (1.2)
Trabecular tumour of the thyroid	1 (0.6)
**Lobe volume (ml), median (i.q.r.)**	58 (28–105)
Unreported, *n*	3
**Pathology**	
Benign	104 (62)
Malignant	65 (38)
Histological thyroiditis*	49 (29)
**VN position within carotid sheath**	
Posterior	125 (84)
Anterior	18 (12)
Posterior to common carotid artery	5 (3.4)
Posterior to internal jugular vein	1 (0.7)
Unreported, *n*	20
**APS^®^ electrode size (mm)**	
2	135 (80)
3	34 (20)
**No. of APS^®^ electrode dislocations**	
0	121 (75)
1	31 (19)
2	5 (3.1)
3	5 (3.1)
Unreported, *n*	7
**Stimulation duration (min), median (i.q.r.)**	45 (40–60)
Unreported, *n*	20

Values are *n* (%) unless otherwise indicated. *Histopathological examination revealed Ewing’s sarcoma of the thyroid gland. WDTC, well differentiated thyroid carcinoma; i.q.r., interquartile range; VN, vagus nerve; APS, automatic periodic stimulation.

Out of the 101 patients, 4 (3.96 per cent) had an intraoperative LOS on the RLN (in three of those cases the signal was not recovered during the intervention), including 1 patient in whom a segment of the nerve had to be sacrificed because of invasion by a neoplasm, resulting in permanent RLN palsy. Postoperative laryngoscopy confirmed unilateral vocal cord dysfunction in these three patients. The two other cases of postoperative RLN palsy were caused by the dissection of the RLN during excision of the thyroid lobe and recovered after 3 and 6 months respectively. In one instance the LOS on the RLN was focal, whereas in the other a global LOS due to excessive traction on the lobe was observed. All four cases of LOS on the RLN are shown in *Table 4*, as well as seven other patients whose measurements at any of the three timings showed an amplitude gap greater than 20 per cent, which was an arbitrary cut-off chosen to highlight potentially consequential decreases in amplitude.

**Table 4:** Patients with notable events (loss of signal at vagus nerve or recurrent laryngeal nerve or decrease greater than 20 per cent of amplitude on the vagus nerve at any of the three timings)

Patient	*ΔV1.0-V1.1*	*ΔV1.2prox-V1.2dist*	*ΔV2prox-V2dist*	LOS at RLN	RLN palsy*	Evolution	Comment
2	−20.48^†^	−4.52	−2.23	No	NA	–	–
5	−9.81	−2.41	−53.19^†^	No	NA	–	–
6	+6.84	−10.84	+4.27	Yes^†^	NA	–	Reversible LOS at RLN
25	−2.34	−5.80	−44.52^†^	No	NA	–	–
30	−4.99	LOS^†^	NA	Yes^†^	Yes^†^	Recuperation 6 months	LOS due to application of electrode. LOS at level of RLN was also observed.
68	+0.82	−3.94	−44.54^†^	No	NA	–	–
72	NR	−24.60^†^	−3.56	No	NA	–	–
74	+32.87	−4.08	−44.20^†^	No	NA	–	–
79	+0.00	−0.44	NA	Yes^†^	Yes^†^	Recuperation 3 months	–
87	+13.69	+1.97	NA	Yes^†^	Yes^†^	Persistent	Resection of RLN with tumour
94	−27.44^†^	−5.86	−1.57	No	NA	–	–

Values are %. *Postoperative laryngoscopy was only performed in cases of significant reductions in amplitudes or in case of vocal hoarseness after surgery. LOS, loss of signal; RLN, recurrent laryngeal nerve; NA, not applicable; NR, not reported. –, no noteworthy elements were reported. ^†^Indicates noteworthy values.

The first surgical step in use of CIONM, the VN dissection, caused a marked and statistically significant increase in signal amplitude (*ΔV1.0-V1.1*) of +30.37 µV (95 per cent c.i. +10.15 to +50.60 µV) (*P* = 0.0035) as indicated in *Table 5*. The authors observed a marked decrease in amplitude of greater than 20 per cent in two patients (shown in *Table 4*).

**Table 5:** Summary of differences in electromyographic signal amplitudes measured at vagus nerve dissection (*ΔV1.0-V1.1*), after automatic periodic stimulation electrode application (*ΔV1.2prox-V1.2dist*), and before automatic periodic stimulation electrode removal (*ΔV2prox-V2dist*)

	*ΔV1.0-V1.1*, before and after VN dissection	*ΔV1.2prox-V1.2dist*, immediately after APS^®^ electrode placement	*ΔV2prox-V2dist*, before electrode removal
No. of nerves	126/169	165/169	162/169
µV, mean (95% c.i.), *P*	+30.37 (+10.15,+50.60), 0.0035	−10.94 (−17.06,−4.82), 0.0005	−18.58 (−28.31,−8.86), 0.0002
Percentage change, mean(s.d.)	+7.95(37.69)	−1.44(5.38)	−2.50(9.59)

VN, vagus nerve; APS, automatic periodic stimulation.

**Fig. 2:**
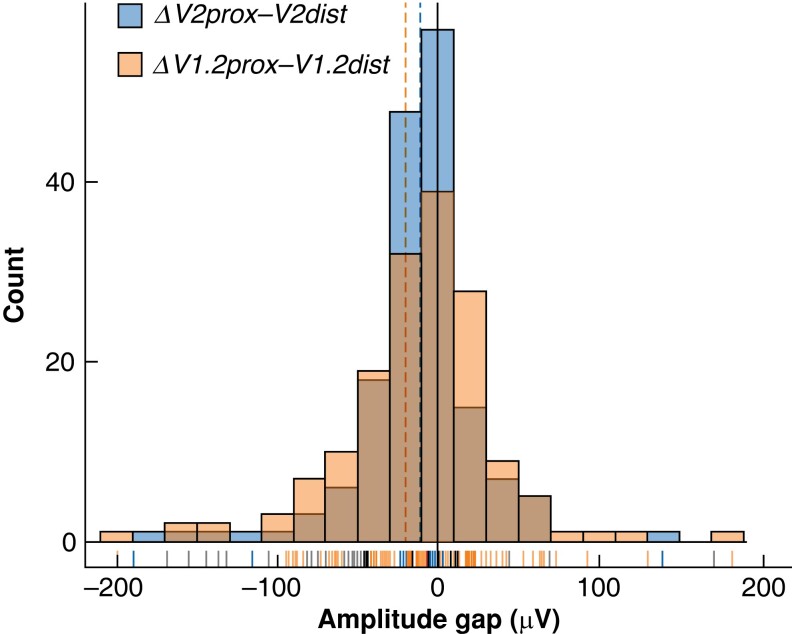
Histogram of the measured differences in electromyographic signal amplitudes after automatic periodic stimulation electrode application (*ΔV1.1prox-V1.2dist*) and before automatic periodic stimulation electrode removal (*ΔV2prox-V2dist*) The dashed lines represent the mean values for each group (−10.94 μV for *ΔV1.1prox-V1.2dist* and −18.58 μV for *ΔV2prox-V2dist*). The graph is calibrated to show values from −200 to +200 µV; some extreme values are therefore not shown. The values for both measurements follow an almost identical Gaussian distribution.

Upon application of the APS^®^ electrode, the measured difference in amplitude (*ΔV1.2prox-V1.2dist*) was a statistically significant decrease of −10.94 µV (95 per cent c.i. −17.06 to −4.82 µV) (*P* = 0.0005) (*Table 5* and *Fig. 2*). One patient had a complete LOS immediately after application of the APS^®^ electrode on to the VN, probably due to excessive traction during the application manoeuvre. The amplitude measured proximally to the electrode was undetectable, but that measured distally to the electrode was approximately 400 µV. Furthermore, another LOS on the ipsilateral RLN was observed during the same operation performed under CIONM after the distal placement of the APS^®^ electrode. The surgery was planned as unilateral thyroid lobectomy, and this event did not result in a two-stage surgery. Postoperative laryngoscopy confirmed a unilateral vocal cord palsy. This patient was excluded from the statistical analysis, given that the extreme values could alter the appreciation of small differences in nerve conduction with CIONM. Only one other nerve displayed a loss of amplitude above 20 per cent at this step of the intervention (shown in *Table 4*).

At the end of the intervention and before the removal of the APS^®^ electrode, the measured difference in amplitude (*ΔV2prox-V2dist*) was a statistically significant decrease of −18.58 µV (95 per cent c.i. −28.31 to −8.86 µV) (*P* = 0.0002) (*Table 5* and *Fig. 2*), which was greater than the amplitude gap measured at the earlier stage of electrode placement upon the VN. Four nerves displayed a decrease in amplitude of over 20 per cent at this stage of the intervention, three more than upon electrode placement (*Table 4*).

**Fig. 3:**
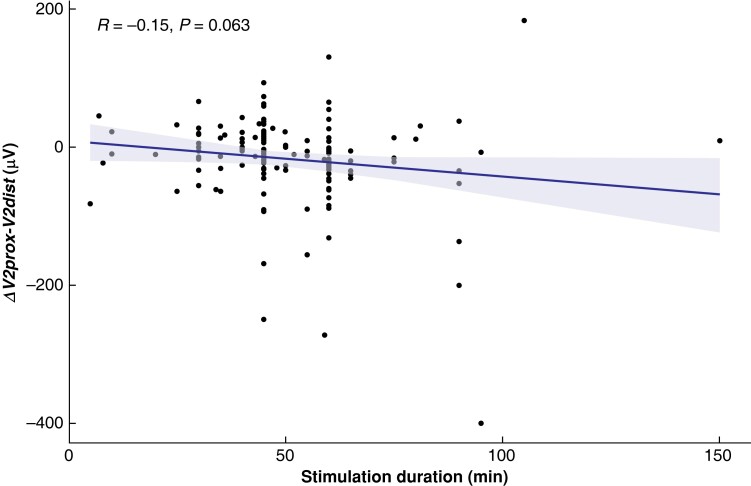
Univariate linear regression of automatic periodic stimulation duration

Once the differences in amplitude were obtained, the authors correlated the observed differences with various demographic, pathological, and surgical characteristics with univariate and multivariate analyses using simple linear regression and multiple linear regression respectively. None of the predictors was found to be associated with an increased amplitude gap at any of the surgical steps. The results are documented in *Tables S1, S2*. The duration of APS^®^ electrode stimulation and the number of APS^®^ electrode dislocations were not found to have a statistically significant association with the amplitude gap measured at *ΔV2prox-V2dist* (*Fig. 3*).

Regarding the systemic effects of VN stimulation, the anaesthesiology team reported a single instance of mild hypotension with a mean blood pressure at 59 mmHg upon APS^®^ electrode activation. This was rapidly reversible when CIONM was stopped. This isolated episode of hypotension did not abort the surgery. No other cardiovascular effects imputable to CIONM were reported in any other patient.

## Discussion

The results of this study point to a statistically significant yet very mild electrophysiological impact on nerve conduction of the VN–RLN axis caused by the use of CIONM in the setting of thyroid and parathyroid surgery. These subtle electrophysiological modifications were not associated with measurable clinical outcomes.

However, the authors report a case of total LOS linked to the traumatic application of the APS^®^ electrode and another case of a marked decrease in amplitude after VN dissection. In both instances, surgery on the affected side was continued using CIONM by placing the APS^®^ electrode below the site of damage. Thus, the concerns over the safety of CIONM are founded. Overall, the benefits regarding the prevention of postoperative RLN palsy outweigh its risks, particularly in patients in whom the RLN is deemed at high risk. Thus, surgeons are advised to be attentive to potential trauma to the VN during dissection and electrode placement.

The rate of postoperative RLN palsy obtained is consistent with the literature^1^, especially as the cases included were at particularly high risk. The continuous EMG recordings provided by CIONM warned of any drop in signal amplitude greater than 50 per cent, motivating us to put an end to any manoeuvre jeopardizing the nerve or even to change approach entirely in the case of sustained alarms^20^.

A marked increase was observed in the amplitude after VN dissection, which can be explained by the removal of connective tissue surrounding the VN, thus improving the transmission of electrical stimulation directly to the VN. The relatively broad standard deviation (37.69 per cent) suggests that, in some cases, the dissection phase may have caused a certain degree of trauma, resulting in a lower EMG wave amplitude. For instance, two patients showed a clear decrease in amplitude above 20 per cent after VN dissection, despite repositioning of the endotracheal tube. Nonetheless, the resulting signal was sufficient to continue surgery with CIONM in all cases.

Upon APS^®^ electrode application to the VN, the authors observed a slight decrease in amplitude, corresponding to a mean of −1.44 per cent with one case of total LOS due to traumatic application of the APS^®^ electrode and one other with an amplitude gap over 20 per cent. An explanation for this small mean decrease in amplitude could be very small VN lesions during APS^®^ electrode placement (in particular, counter-traction when the VN is deeply located in the neck) or light compression of the VN by the electrode. Measurements made before the removal of the APS^®^ electrode revealed a greater decrease of −2.50 per cent, with four nerves showing a proximo-distal amplitude gap over 20 per cent. This result is marginally higher than that for the previous event of the first APS^®^ electrode application, and it is hypothesized that this difference may be linked to prolonged light compression of the VN or a decrease in the segment’s conductivity caused by the continuous stimulation throughout the intervention.

It should be pointed out that in seven instances, including the case of total LOS on the VN, an amplitude gap greater than 20 per cent was measured. Nonetheless, despite being statistically significant, the measured changes in proximo-distal stimulation at the level of the APS^®^ electrode upon application and removal of −1.44 per cent and −2.5 per cent respectively are very small. It seems highly unlikely that the minor decreases observed in the majority of cases may lead to an altered functional outcome or visible change upon laryngoscopic examination of the vocal cords, as VN stimulation proximal to the electrode was sufficient in all cases for postoperative laryngoscopy to be unwarranted (median of 659 (i.q.r. 505–944) µV; 160 patients)^36,37^.

Multiple regression analysis of the documented demographic and surgical predictors did not reveal any noteworthy associations. The duration of stimulation seemed to be associated with a difference in conduction, as shown by the univariate regression, although a statistically significant association was not found (*P* = 0.06). This is consistent with the authors' previous observation that the differences in measured amplitude were greater at the end of the intervention, before the removal of the APS^®^ electrode, but, when adjusted with the other predictors in the multivariate analysis, this association did not persist (estimated loss of −0.46 µV (95 per cent c.i. −1.07 to 0.15 µV) per additional minute of stimulation; *P* = 0.140). In their prospective multicentre study, Phelan *et al*.^38^ reached a similar conclusion with regard to the innocuity of the duration of stimulation. It is interesting to note that the number of unintentional dislocations of the APS^®^ electrode was not associated with decreased conduction, which suggests an adequate atraumatic design of the electrode.

One limitation of this study was the small sample size and limited statistical power, especially considering that LOS on the VN is a rare event. Moreover, patients were not systematically subjected to postoperative laryngoscopy when they did not display significant intraoperative LOS, as it has been shown with negative predictive values (NPVs) of greater than 99 per cent that a good EMG recording of the VN–RLN axis at the end of surgery is sufficient to rule out clinically significant vocal cord dysfunction^36,37,39^.

When comparing the measured amplitudes, the study was limited by the fact that it is difficult to compare the proximo-distal amplitudes measured directly after application of the APS^®^ electrode with those acquired before its removal because the events were chronologically separated, exposing any pairing of the values to potential external bias caused by the effects of residual curarization and endotracheal tube displacement.

Another limitation was that the latency of the measured signals was not reported. However, as latency is dependent upon the distance from the stimulation site to the recorded site, and as the primary outcome was a measurement at different distances (above *versus* below the APS^®^ electrode placement), latency was considered not to be a good marker of nerve damage. Moreover, as differences in the measured latency in this setting are usually very small (an increase of only 10 per cent is sufficient for the system to trigger an alarm), the number of patients to achieve statistical significance would be much higher than the authors could manage to recruit. In addition, Brauckhoff *et al*.^33^ suggested that an increased measured latency would not by itself be an accurate reflection of a decrease in nerve conduction velocity due to RLN damage, as the neuromuscular junction alone may account for up to 50 per cent of the measured latency.

In the authors' experience, CIONM can be used in patients with an LOS on the VN, by placing the APS^®^ electrode distally to the damaged site; then surgery in the ipsilateral central compartment could be pursued as planned. If LOS proximal to the site of electrode placement persists at the end of the first side in a planned bilateral procedure, the surgery is stopped after the first side. To date, four cases of LOS on the VN have been reported (one case in this study and three cases in the 2018 paper^20^) that have all recovered and allowed the authors to continue with the second side, when necessary, using a safe ‘staged thyroidectomy’ protocol. For these reasons, the INMSG guidelines should be updated to clearly state that VN stimulation after thyroid lobe extraction (V2) should be obtained with IONM stimulation above the site of CIONM electrode placement if CIONM is used. The NPV of a good V2 IONM signal with respect to the prediction of RLN function would still be higher if such a protocol was used. In other words, some RLN palsies described after the measurement of a good V2 signal are due to VN lesions occurring proximal to the measured V2 site.

## Supplementary Material

zrad039_Supplementary_Data

## Data Availability

The data that support the findings of this study are available from the corresponding author, T.M., upon reasonable request.

## References

[1] Hayward NJ , Grodski S, Yeung M, Johnson WR, Serpell J. Recurrent laryngeal nerve injury in thyroid surgery: a review. ANZ J Surg 2013;83:15–21 22989215 10.1111/j.1445-2197.2012.06247.x

[2] Thomusch O , Machens A, Sekulla C, Ukkat J, Lippert H, Gastinger I et al Multivariate analysis of risk factors for postoperative complications in benign goiter surgery: prospective multicenter study in Germany. World J Surg 2000;24:1335–1341 11038203 10.1007/s002680010221

[3] Bellantone R , Lombardi CP, Bossola M, Boscherini M, De Crea C, Alesina P et al Total thyroidectomy for management of benign thyroid disease: review of 526 cases. World J Surg 2002;26:1468–1471 12360381 10.1007/s00268-002-6426-1

[4] Efremidou EI , Papageorgiou MS, Liratzopoulos N, Manolas KJ. The efficacy and safety of total thyroidectomy in the management of benign thyroid disease: a review of 932 cases. Can J Surg 2009;52:39–44 19234650 PMC2637645

[5] Duclos A , Peix JL, Colin C, Kraimps JL, Menegaux F, Pattou F et al Influence of experience on performance of individual surgeons in thyroid surgery: prospective cross sectional multicentre study. BMJ 2012;344:d8041 22236412 10.1136/bmj.d8041 PMC3256252

[6] Macias AA , Eappen S, Malikin I, Goldfarb J, Kujawa S, Konowitz PM et al Successful intraoperative electrophysiologic monitoring of the recurrent laryngeal nerve, a multidisciplinary approach: the Massachusetts Eye and Ear Infirmary monitoring collaborative protocol with experience in over 3000 cases. Head Neck 2016;38:1487–1494 27062311 10.1002/hed.24468

[7] Nouraei SAR , Allen J, Kaddour H, Middleton SE, Aylin P, Darzi A et al Vocal palsy increases the risk of lower respiratory tract infection in low-risk, low-morbidity patients undergoing thyroidectomy for benign disease: a big data analysis. Clin Otolaryngol 2017;42:1259–1266 28616866 10.1111/coa.12913

[8] Heitmiller RF , Tseng E, Jones B. Prevalence of aspiration and laryngeal penetration in patients with unilateral vocal fold motion impairment. Dysphagia 2000;15:184–187 11014880 10.1007/s004550000026

[9] Smith E , Taylor M, Mendoza M, Barkmeier J, Lemke J, Hoffman H. Spasmodic dysphonia and vocal fold paralysis: outcomes of voice problems on work-related functioning. J Voice 1998;12:223–232 9649078 10.1016/s0892-1997(98)80042-8

[10] Kern KA . Medicolegal analysis of errors in diagnosis and treatment of surgical endocrine disease. Surgery 1993;114:1167–1173; discussion 1173–1174 8256224

[11] Angelos P . Ethical and medicolegal issues in neuromonitoring during thyroid and parathyroid surgery: a review of the recent literature. Curr Opin Oncol 2012;24:16–21 22051523 10.1097/CCO.0b013e32834cd596

[12] Polistena A , Di Lorenzo P, Sanguinetti A, Buccelli C, Conzo G, Conti A et al Medicolegal implications of surgical errors and complications in neck surgery: a review based on the Italian current legislation. Open Med (Wars) 2016;11:298–306 28352812 10.1515/med-2016-0058 PMC5329845

[13] Schneider R , Randolph G, Dionigi G, Barczyński M, Chiang F-Y, Triponez F et al Prospective study of vocal fold function after loss of the neuromonitoring signal in thyroid surgery: the International Neural Monitoring Study Group’s POLT study. Laryngoscope 2016;126:1260–1266 26667156 10.1002/lary.25807

[14] Schneider R , Sekulla C, Machens A, Lorenz K, Thanh PN, Dralle H. Postoperative vocal fold palsy in patients undergoing thyroid surgery with continuous or intermittent nerve monitoring. BJS 2015;102:1380–1387 10.1002/bjs.9889 26333134

[15] Schneider R , Sekulla C, Machens A, Lorenz K, Thanh PN, Dralle H. Dynamics of loss and recovery of the nerve monitoring signal during thyroidectomy predict early postoperative vocal fold function. Head Neck 2016;38:E1144–E1151 26331940 10.1002/hed.24175

[16] Lamadé W , Ulmer C, Seimer A, Molnar V, Meyding-Lamadé U, Thon K-P et al A new system for continuous recurrent laryngeal nerve monitoring. Minim Invasive Ther Allied Technol 2007;16:149–154 17573619 10.1080/13645700701383241

[17] Schneider R , Randolph GW, Sekulla C, Phelan E, Thanh PN, Bucher M et al Continuous intraoperative vagus nerve stimulation for identification of imminent recurrent laryngeal nerve injury. Head Neck 2013;35:1591–1598 23169450 10.1002/hed.23187

[18] Schneider R , Randolph GW, Barczynski M, Dionigi G, Wu C-W, Chiang F-Y et al Continuous intraoperative neural monitoring of the recurrent nerves in thyroid surgery: a quantum leap in technology. Gland Surg 2016;5:607–616 28149807 10.21037/gs.2016.11.10 PMC5233836

[19] Schneider R , Bures C, Lorenz K, Dralle H, Freissmuth M, Hermann M. Evolution of nerve injury with unexpected EMG signal recovery in thyroid surgery using continuous intraoperative neuromonitoring. World J Surg 2013;37:364–368 23188536 10.1007/s00268-012-1853-0

[20] Marin Arteaga A , Peloni G, Leuchter I, Bedat B, Karenovics W, Triponez F et al Modification of the surgical strategy for the dissection of the recurrent laryngeal nerve using continuous intraoperative nerve monitoring. World J Surg 2018;42:444–450 28986621 10.1007/s00268-017-4277-z

[21] Kandil E , Mohsin K, Murcy MA, Randolph GW. Continuous vagal monitoring value in prevention of vocal cord paralysis following thyroid surgery. Laryngoscope 2018;128:2429–2432 29481696 10.1002/lary.27144

[22] Schneider R , Machens A, Sekulla C, Lorenz K, Elwerr M, Dralle H. Superiority of continuous over intermittent intraoperative nerve monitoring in preventing vocal cord palsy. Br J Surg 2021;108:566–573 34043775 10.1002/bjs.11901

[23] Chiang F-Y , Lu I-C, Kuo W-R, Lee K-W, Chang N-C, Wu C-W. The mechanism of recurrent laryngeal nerve injury during thyroid surgery—the application of intraoperative neuromonitoring. Surgery 2008;143:743–749 18549890 10.1016/j.surg.2008.02.006

[24] Schneider R , Machens A, Randolph GW, Kamani D, Lorenz K, Dralle H. Opportunities and challenges of intermittent and continuous intraoperative neural monitoring in thyroid surgery. Gland Surg 2017;6:537–545 29142846 10.21037/gs.2017.06.08 PMC5676169

[25] Bacuzzi A , Dionigi G, Del Bosco A, Cantone G, Sansone T, Di Losa E et al Anaesthesia for thyroid surgery: perioperative management. Int J Surg 2008;6:S82–S85 19195946 10.1016/j.ijsu.2008.12.013

[26] Lu I-C , Chu K-S, Tsai C-J, Wu C-W, Kuo W-R, Chen H-Y et al Optimal depth of NIM EMG endotracheal tube for intraoperative neuromonitoring of the recurrent laryngeal nerve during thyroidectomy. World J Surg 2008;32:1935–1939 18392652 10.1007/s00268-008-9549-1

[27] Tsai C-J , Tseng K-Y, Wang F-Y, Lu I-C, Wang H-M, Wu C-W et al Electromyographic endotracheal tube placement during thyroid surgery in neuromonitoring of recurrent laryngeal nerve. Kaohsiung J Med Sci 2011;27:96–101 21421197 10.1016/j.kjms.2010.08.002 PMC11916461

[28] Barber SR , Liddy W, Kyriazidis N, Cinquepalmi M, Lin BM, Modi R et al Changes in electromyographic amplitudes but not latencies occur with endotracheal tube malpositioning during intraoperative monitoring for thyroid surgery: implications for guidelines. Laryngoscope 2017;127:2182–2188 27861939 10.1002/lary.26392

[29] Terris DJ , Chaung K, Duke WS. Continuous vagal nerve monitoring is dangerous and should not routinely be done during thyroid surgery. World J Surg 2015;39:2471–2476 26138874 10.1007/s00268-015-3139-9

[30] Groves DA , Brown VJ. Vagal nerve stimulation: a review of its applications and potential mechanisms that mediate its clinical effects. Neurosci Biobehav Rev 2005;29:493–500 15820552 10.1016/j.neubiorev.2005.01.004

[31] Mertens A , Raedt R, Gadeyne S, Carrette E, Boon P, Vonck K. Recent advances in devices for vagus nerve stimulation. Expert Rev Med Devices 2018;15:527–539 30071175 10.1080/17434440.2018.1507732

[32] Ben-Menachem E . Vagus nerve stimulation, side effects, and long-term safety. J Clin Neurophysiol 2001;18:415–418 11709646 10.1097/00004691-200109000-00005

[33] Brauckhoff K , Vik R, Sandvik L, Heimdal J-H, Aas T, Biermann M et al Impact of EMG changes in continuous vagal nerve monitoring in high-risk endocrine neck surgery. World J Surg 2016;40:672–680 26678490 10.1007/s00268-015-3368-y PMC4746223

[34] Chiang F-Y , Lee K-W, Chen H-C, Chen H-Y, Lu I-C, Kuo W-R et al Standardization of intraoperative neuromonitoring of recurrent laryngeal nerve in thyroid operation. World J Surg 2010;34:223–229 20020124 10.1007/s00268-009-0316-8

[35] Randolph GW , Dralle H, Abdullah H, Barczynski M, Bellantone R, Brauckhoff M et al Electrophysiologic recurrent laryngeal nerve monitoring during thyroid and parathyroid surgery: international standards guideline statement. Laryngoscope 2011;121:S1–S16 21181860 10.1002/lary.21119

[36] Thomusch O , Sekulla C, Machens A, Neumann H-J, Timmermann W, Dralle H. Validity of intra-operative neuromonitoring signals in thyroid surgery. Langenbecks Arch Surg 2004;389:499–503 14722777 10.1007/s00423-003-0444-9

[37] Genther DJ , Kandil EH, Noureldine SI, Tufano RP. Correlation of final evoked potential amplitudes on intraoperative electromyography of the recurrent laryngeal nerve with immediate postoperative vocal fold function after thyroid and parathyroid surgery. JAMA Otolaryngol Head Neck Surg 2014;140:124–128 24384927 10.1001/jamaoto.2013.6139 PMC4597471

[38] Phelan E , Schneider R, Lorenz K, Dralle H, Kamani D, Potenza A et al Continuous vagal IONM prevents recurrent laryngeal nerve paralysis by revealing initial EMG changes of impending neuropraxic injury: a prospective, multicenter study. Laryngoscope 2014;124:1498–1505 24307596 10.1002/lary.24550

[39] Calò PG , Pisano G, Medas F. Intraoperative recurrent laryngeal nerve monitoring in thyroid surgery: is it really useful? Clin Ter 2013;164:e193–e198 23868637 10.7417/CT.2013.1567

