# Supplementary material for: Safety of continuous intraoperative vagus nerve neuromonitoring during thyroid surgery
Source: BJS Open. 2023 Jun 8;7(3):zrad039. doi: 10.1093/bjsopen/zrad039 (PMC10249491; doi:10.1093/bjsopen/zrad039)
Supplement: zrad039_Supplementary_Data [file zrad039_supplementary_data.docx]

Safety of Continuous Intra-Operative Vagus Nerve Neuromonitoring during Thyroid Surgery

Timothy Mathieson^1^, Wedali Jimaja^1^, Frédéric Triponez^1^, Marc Licker^2^, Wolfram Karenovics^1^, Petra Makovac^1^, Mirza Muradbegovic^1^, Valentina Belfontali^1^, Benoît Bédat^1^ and Marco Stefano Demarchi^1^

^1^Department of Thoracic and Endocrine Surgery and Faculty of Medicine, University Hospitals of Geneva, 4 Rue Gabrielle Perret-Gentil, 1211 Geneva, Switzerland;

^2^Department Anesthesiology and Faculty of Medicine, University hospitals of Geneva, 4 Rue Gabrielle Perret-Gentil, 1211 Geneva, Switzerland

***** Correspondence: timothy.mathieson@hcuge.ch (T.M.); Tel.: +41 (0)22 372 78 62

**Supplementary Materials - Index**

| **Supplementary Figures and Tables** |  |
| --- | --- |
| Table S1 | *pag. 2* |
| Table S2 | *pag. 2* |
|  |  |

**Supplementary Figures and Tables**

**Table S1**

| ΔV1.2prox-V1.2dist | | | |
| --- | --- | --- | --- |
| Predictors | Estimates | CI | p |
| (Intercept) | -44.91 | -90.51 — 0.69 | 0.054 |
| Age | 0.10 | -0.40 — 0.61 | 0.691 |
| BMI | 0.66 | -0.54 — 1.87 | 0.280 |
| Lobe volume (ml) | 0.09 | 0.01 — 0.18 | **0.035** |
| Histological thyroiditis | 6.76 | -11.10 — 24.62 | 0.456 |
| Central neck dissection^1^ | 6.26 | -11.68 — 24.20 | 0.491 |
| Lateral neck dissection^1^ | 25.34 | -56.04 — 106.72 | 0.539 |
| Revision surgery | -7.45 | -65.44 — 50.53 | 0.800 |
| VN in anterior position within carotid sheath^2^ | -1.70 | -22.65 — 19.24 | 0.872 |
| VN posterior to CCA ^2^ | 32.12 | -4.25 — 68.49 | 0.083 |
| VN posterior to IJV^2^ | 15.10 | -65.71 — 95.91 | 0.712 |
| Observations | 144 |  |  |
| R^2^ / R^2^ adjusted | 0.070 / -0.000 |  |  |
|  |  |  |  |
| ^1^ Reference = no neck dissection  ^2^ Reference = VN in posterior position |  |  |  |

***Table S1:.*** *Summary of the multiple linear regression assessing the link between ΔV2prox-V2dist and various demographic and surgical predictors*

*BMI = body mass index ; VN = vagus nerve ; CCA = common carotid artery ; IJV = internal jugular vein*

**Table S2**

| ΔV2prox-V2dist | | | |
| --- | --- | --- | --- |
| Predictors | Estimates | CI | p |
| (Intercept) | 19.17 | -63.23 — 101.56 | 0.646 |
| Age | -0.06 | -0.90 — 0.78 | 0.890 |
| BMI | -0.14 | -2.06 — 1.79 | 0.889 |
| Lobe volume (ml) | -0.00 | -0.14 — 0.13 | 0.946 |
| Histological thyroiditis | -3.53 | -32.80 — 25.75 | 0.812 |
| Central neck dissection^1^ | -0.70 | -33.24 — 31.83 | 0.966 |
| Lateral neck dissection^1^ | -1.81 | -132.05 — 128.44 | 0.978 |
| Revision surgery | 36.08 | -56.87 — 129.02 | 0.444 |
| VN in anterior position within carotid sheath^2^ | -2.08 | -36.43 — 32.27 | 0.905 |
| VN posterior to CCA ^2^ | -53.41 | -111.60 — 4.78 | 0.072 |
| VN posterior to IJV^2^ | -13.41 | -142.89 — 116.07 | 0.838 |
| APS® electrode activation (min) | -0.46 | -1.07 — 0.15 | 0.140 |
| APS® electrode dislocations (n) | -3.40 | -19.54 — 12.75 | 0.678 |
| Observations | 143 |  |  |
| R^2^ / R^2^ adjusted | 0.037 / -0.036 |  |  |
|  |  |  |  |
| ^1^ Reference = no neck dissection  ^2^ Reference = VN in posterior position |  |  |  |

***Table S2:.*** *Summary of the multiple linear regression assessing the link between ΔV2prox-V2dist and various demographic and surgical predictors*

*BMI = body mass index ; VN = vagus nerve ; CCA = common carotid artery ; IJV = internal jugular vein ; APS = automatic periodic stimulation*
